# Leucine (and lysine) increased plasma levels of the satiety hormone cholecystokinin (CCK), and phenylalanine of the incretin glucagon-like peptide 1 (GLP-1) after oral gavages in pigs

**DOI:** 10.1093/jas/skad175

**Published:** 2023-05-26

**Authors:** Maximiliano Müller, Chenjing Xu, Marta Navarro, Nuria Elias Masiques, Alan Tilbrook, Robert van Barneveld, Eugeni Roura

**Affiliations:** Centre for Nutrition and Food Sciences, Queensland Alliance for Agriculture and Food Innovation, The University of Queensland, Brisbane, Queensland 4072, Australia; Centre for Nutrition and Food Sciences, Queensland Alliance for Agriculture and Food Innovation, The University of Queensland, Brisbane, Queensland 4072, Australia; Centre for Nutrition and Food Sciences, Queensland Alliance for Agriculture and Food Innovation, The University of Queensland, Brisbane, Queensland 4072, Australia; Laboratory of Animal Nutrition and Animal Product Quality (LANUPRO), Department of Animal Sciences and Aquatic Ecology, Ghent University, Ghent, Flanders 339000, Belgium; Centre for Animal Science, Queensland Alliance for Agriculture and Food Innovation and the School of Veterinary Science, The University of Queensland, Brisbane, Queensland 4072, Australia; SunPork Group, Brisbane, Queensland 4009, Australia; Centre for Nutrition and Food Sciences, Queensland Alliance for Agriculture and Food Innovation, The University of Queensland, Brisbane, Queensland 4072, Australia

**Keywords:** amino acid, blood, cholecystokinin, glucagon-like peptide 1, oral gavage, pig

## Abstract

Excess dietary amino acids (AA) has been associated with reduced feed intake, increased satiation, and extended satiety in pigs. Recent ex vivo studies suggested that satiety peptide cholecystokinin (CCK) and insulinotropic glucagon-like peptide 1 (GLP-1), mediated the anorexigenic or insulinotropic effects of Lys, Glu, Phe, Ile, and Leu. However, the ex vivo model limitations require validation in vivo. The aim of the present study was to assess the effect of orally administered AA in vivo in pigs. It was hypothesized that oral Lys, Ile, and Leu have an anorexigenic effect via CCK, while Glu and Phe have an insulinotropic effect increasing circulating levels of GLP-1. Eight entire male pigs (Landrace × Large White) of 18.23 ± 1.06 kg of body weight were administered an oral gavage of water (control) or a 3 mmol/kg of Glu, Ile, Leu, Lys, Phe, or glucose (positive control for GLP-1 release) following an overnight fasting during 5 consecutive days using an incomplete latin square design. Blood samples were collected from the jugular vein before (−5 min, baseline value) and after the gavage (5, 15, 30, 60 and 90 min) to assess CCK and GLP-1 plasma levels. Pigs administered the oral gavage of Leu (*P* < 0.05), or Lys (*P* < 0.1) had increased levels of plasma CCK from 0 to 90 min post-gavage when compared to the control. A strong association (*P* < 0.001) was observed between GLP-1 plasma levels with Phe intake. The impact was significant starting 30 min post-gavage and was sustained until the end of the experiment (90 min post-gavage). Glucose administration increased GLP-1 early after the intake at the 5 min mark (*P* < 0.1). A positive correlation (*P* < 0.05, *r* = 0.89) driven by the impact of Phe at the 60 to 90 min post-gavage was identified between CCK and GLP-1 indicating feedback mechanisms between proximal and distal small intestine. In conclusion, oral gavages of Leu and Lys increased anorexigenic hormone CCK plasma levels in pigs. Phe caused a significant long-lasting increase in incretin GLP-1 plasma levels. Blood CCK and GLP-1 concentrations in Phe gavaged pigs were positively correlated indicating a potential feedback mechanism between proximal (CCK) and distal (GLP-1) small intestine. The present results are compatible with the known anorexigenic effects of excess dietary Leu and Lys, and the insulinotropic effect of Phe in pigs. These results demonstrate the relevance of accurate feed formulation practices particularly in post weaning pigs.

## Teaser

The administration of Lys, Leu, Ile, Phe, or Glu by oral gavage resulted in Leu and Lys increasing circulating the satiety hormone CCK, and Phe stimulating the release of the incretin GLP-1. The effect of Phe on GLP-1 was sustained beyond 90 min and triggered a positive correlation with CCK indicating the likely existence of feedback mechanisms between proximal and distal regions of the small intestine. These results are relevant to pig feed formulation practices.

## Introduction

Amino acids (AA) play a pivotal role on the modulation of appetite in pigs (Roura and Navarro, 2018; Müller et al., 2021). In particular, the effect of some of the most limiting essential AA including Lys, Met, Thr, Trp, Leu, or Arg on feed intake has been previously uncovered (Edmonds and Baker, 1987; Edmonds et al., 1987). Recently some of the key gastrointestinal tract (GIT) molecular and cellular mechanisms mediating the appetite modulating effects of AA have been described in pigs. The GIT senses the arrival of dietary AA mainly through transmembrane receptors present in the intestinal epithelium associated to enteroendocrine cells (EEC) (Roura et al., 2019). The activation of EEC results in the release of gut peptides that regulate feed intake by interacting with hypothalamic neurons (area postrema) via blood or by stimulating the vagal nerve (Müller et al., 2021). Of the 20 proteinogenic AA, Leu, Ile, Phe, and Glu were described as some of the strongest secretagogues of appetite-suppressing peptide cholecystokinin (CCK) and/or the insulinotropic hormone glucagon-like peptide 1 (GLP-1) using a porcine ex vivo model of primary intestinal tissue cultures (Feng et al., 2019, Tian et al., 2019, Müller et al., 2022a). Dietary Lys has been reported to increase the gene expression of CCK along the small intestine (Yin et al., 2017). In addition, the existence of feedback mechanisms between proximal and distal segments of the GIT are essential to understand the impact of AA and other nutrients on appetite. EEC respond to gut contents but also to circulating levels of gut peptides and bacterial metabolites released along the GIT (Furness et al., 2013). Thus, the value of excised intestinal tissues such as in ex vivo methods is limited due to the lack of feedback mechanisms, thus requiring validation using integral systems (i.e., live animals).

In live pigs, Müller and co-workers (2022b) studied the impact of oral free AA solutions on feeding behavior showing that an oral gavage of Lys elicited early satiation and prolonged satiety in young pigs. In addition, Leu, or Ile also prolonged satiety (Müller et al., 2022b). These findings are relevant in the context of standard formulation practices where low levels of crude protein supplemented with high concentrations limiting AA (as crystalline AA) are commonly used, particularly during the post weaning period (Wang et al., 2018). However, the link between plasma levels of gut peptides and feeding behavior in pigs have not been addressed in a systematic approach to date. The aim of this study was to investigate the effect of Lys, Leu, Ile, Phe, and Glu on the blood kinetics of the anorexigenic hormones CCK and the insulinotropic GLP-1 in young pigs. It was hypothesized that oral AA solutions would significantly increase CCK and/or GLP-1 blood levels on a time-dependent manner compatible with the onset of satiety, and prolonged satiation in live pigs.

## Materials and Methods

### Animal ethics

The study was conducted under veterinary supervision and approved by The University of Queensland Animal Ethics Committee (Animal Ethics Certificate: CNFS/568/16).

### Animals, housing, and diet

Eight entire male pigs (Landrace × Large White; body weight = 18.23 ± 1.06 kg) were individually housed in slatted floor pens (1.7 m × 1.2 m), kept with 12 h of light (intensity of 40 to 60 lux) at a room temperature between 23 and 24 °C at the Herston Medical Research Centre at The University of Queensland (Herston Campus, Queensland, Australia). Pigs had ad libitum access to feed and water throughout the experiment, unless otherwise stated. The experimental diet was formulated with a higher crude protein (25%) content than standard commercial feeds to cover nutritional requirements without using synthetic AA (Table 1). Lethabarb (162.5 mg/kg) (Milperra, New South Wales, Australia) was administered intravenously at the end of the experiment to humanely sacrifice the pigs.

**Table 1.** Composition of the experimental diet (as fed basis)

Item	Diet
Ingredients, %	
Wheat	60.80
Soya bean full	16.00
Blood meal	3.00
Meat meal	6.55
Fish meal	4.25
Chocolate milk powder	5.00
Single cell protein	2.50
Vegetable oil	1.50
Salt	0.15
Choline chloride 60%	0.04
Vitamin and mineral premix^1^	0.20
Calculated nutrient content, %	
Crude protein	24.97
Digestible energy (MJ/kg)	15.25
Calcium	1.18
Phosphorus	0.86
Lysine	1.40
Methionine	0.44
Threonine	0.94
Tryptophan	0.28
Met/Lys	0.31
(Met + Cys)/Lys	0.60
Trp/Lys	0.20
Thr/Lys	0.67
Analyzed composition^2^, %	
Crude protein	24.65
Moisture	8.20
Ash	5.27
Crude fiber	2.56
Ether extract	6.95
Lysine	1.32
Methionine	0.41
Threonine	0.93
Tryptophan	0.29
Glycine	1.31
Histidine	0.73
Arginine	1.50
Alanine	1.41
Tyrosine	0.69
Valine	1.19
Serine	1.11
Phenylalanine	1.25
Isoleucine	0.87
Leucine	1.83
Glutamic acid	4.45
Proline	1.67
Hydroxyproline	0.27
Aspartic acid	2.05

^1^Premix composition (ad-fed basis): vitamin A, 10,000 IU/kg; vitamin D3, 1,800 IU/kg; vitamin E, 100 mg/kg; vitamin K3, 5 mg/kg; vitamin B1, 3 mg/kg; vitamin B2, 6 mg/kg; niacin, 30 mg/kg; pantothenic acid, 30 mg/kg; pyridoxine, 4 mg/kg; biotin, 0.3 mg/kg; folic acid, 2.5 mg/kg; vitamin B12, 0.04 mg/kg; iron, 100 mg/kg; iodine, 0.7 mg/kg; manganese, 45 mg/kg; selenium, 0.3 mg/kg; zinc, 120 mg/kg; cobalt, 0.3 mg/kg; copper, 10 mg/kg.

^2^Based on laboratory proximal and AA analysis.

### Habituation, jugular vein catheterization and post-surgery recovery

Following an overnight fasting of 13 h all the pigs in the study were applied a jugular catheter using surgical procedures following the procedures from Pluschke et al. (2016). In brief, the procedure included a habituation to human handling to minimize the stress consisting of gently rubbing, patting, and scratching the neck area twice for 10 min before the start of the catheterization when pigs were sedated with an intramuscular injection of Ketamine (5 mg/kg) and Xylazil (0.35 mg/kg) and transferred to the surgery room. Pigs were fully sedated using isoflurane (4%) through an anesthetic facemask inserted in the airway (5.5 mm endotracheal tube) connected to an anesthetic machine and placed in dorsal recumbency position to ensure continuous and steady administration of the chemical at 1.5 to 3% during the surgical procedure. Pig’s heart and breathing rate, and blood oxygen levels were constantly monitored during the anesthesia. After reaching the point of complete anesthesia pigs’ neck were shaved and scrubbed. A linear incision of approx. 5 cm was made next to the lateral sternomandibularis muscle and a blunt dissection was used to expose the external jugular vein. Two ligatures were placed on the cranial and caudal side of the exposed section of the external jugular vein to keep the vessel in place for the insertion of a central venous catheter. A ligature with monosyn 3/0 around the jugular vein followed to safely fix the catheter in place. The patency of the catheters was checked after the ligation. The incisions were sutured with monosyn, covered with sterile surgical dressing and the catheter fixed to the skin with the same suture. To completely stabilize the catheter and avoid potential external disruptions (such as rubbing, self-inflicted scratching, etc.), sterile adhesive tape (Elastoplast) was used around the neck including gluing (with superglue). A single intramuscular injection of Meloxicam (0.3 mg/kg) and Butorphanol (0.2 mg/kg) coupled with a local anesthetic (Lidocaine, 20 mg/L) infused in the incision site were provided after surgery to reduce the discomfort of the pigs. In addition, a single intramuscular dose of tulathromycin (Draxxin, 2.5 mg/kg) was administered as antibiotic cover.

Following the intervention, pigs were left with oxygen, and monitored until signs of recovery, such as vocalization and leg movements, appeared. Pigs were then transferred back to their pens and monitored until full recovery from the anesthesia before the administration of the first round of oral gavages. Pigs were deemed to be fully recovered when displaying normal behavior including freely moving around the pen, sniffing, drinking, and eating. Full recovery occurred after around 48 h when feed intake levels was above 90% of that shown before surgery. Patency of catheters was checked once a day by the administration of 3 ml saline solution followed by 3 ml heparinized saline solution (100 IU) throughout the recovery period. No adverse events were observed during the surgeries.

### Oral gavage procedure

The oral gavage procedure was performed as previously described by Müller et al. (2022b). In brief, animals were fasted overnight (13 h) before receiving an oral gavage with water (negative control), AA (Lys, Ile, Leu, Glu, or Phe in their l-isomer forms) or glucose (positive control) at a dose of 3 mmol/kg (between 7.8 and 9.9 g based on molecular weight) (Bulk Supplements, Nevada, USA). The AA doses provided were equivalent to daily consumptions by pigs of similar age under commercial practices and the weight on the day of surgery (48 h prior to the first gavage). Prior to the start of the procedure, pigs were moved to a separate room and light anesthesia with isoflurane (1 to 3%) was applied using mask ventilation. When the first signs of the anesthesia started to appear, the pigs were placed on a table with their heads lifted slightly and a 60 ml syringe and a 25 cm long plastic extension tube with soft edges were used to provide the oral gavage. Pigs remained under observation for 2 to 3 min to check for any abnormalities following the procedure. The animals were then returned to their pens for the subsequent blood sampling for the measurement of plasma gut hormone (CCK and GLP-1) levels. The process was repeated on 5 consecutive days to allow for the testing of all treatments in a statistical relevant number of pigs and minimize the number of days that the animals were catheterized to prevent potential harm and/or bacterial contamination. No adverse events were observed during the procedures.

### Blood collection

**Figure 1.**
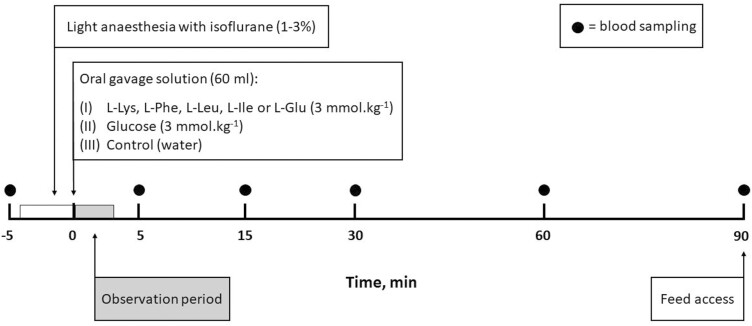
Study protocol for the collection of blood samples in orally gavaged pigs. At *t* = −5 min, baseline blood samples were collected. Immediately after the first blood, sampling light anesthesia with isoflurane was applied by mask ventilation. At *t* = 0, oral gavages with water (control) or 3 mmol/kg solutions of glucose (GLP-1 positive control), Glu, Ile, Leu, Lys, or Phe were administered with a 25 cm plastic extension tube. Following a 2 to 3 min period of observation after the gavage, animals were placed back into their corresponding pen. For the next 90 min after the gavage, blood samples were collected at *t* = 5, 15, 30, 60, and 90 min. At *t* = 90, pigs had ad libitum access to feed again.

Blood samples were collected after overnight fasting following the procedures described by Pluschke and co-workers (2018). Figure 1 shows the timeline of blood sampling including collections before the gavage (time 0 = baseline values) and at 5-, 15-, 30-, 60- and 90-min post-gavage. Before each sampling, 1 ml of blood was discarded to avoid potential heparin contamination. Two ml of blood were collected using a 3 ml syringe and immediately transferred into a pre-chilled P800 vacutainer containing proteases inhibitors. To avoid the hemolysis of samples, blood was retrieved slowly from the catheters and placed carefully in the corresponding vacutainer. Following every sampling, 3 ml of saline solution (0.9%) followed by 1 ml of heparinized saline solution (4 IU) were administered to the pigs to provide fluid restoration and prevent clotting, respectively. After the last blood sampling and final administration of heparinized saline solution (100 IU, to ensure patency of the catheter), the feeders were placed back in each pen. Animals had access to the feed for approx. 9.5 h before the feeders were retrieved again to allow for an overnight fasting prior to the next sampling day.

### Analytical procedures

Blood samples were centrifuged at 3,000 rpm (4 °C) for 10 min within 1 h of collection and the plasma aliquoted in eppendorf tubes before being frozen at −80 °C for hormone analysis. Plasma samples were analyzed for total CCK using a Porcine Cholecystokinin ELISA kit (MBS264395, MyBioSource, San Diego, California, USA). Inter-assay coefficient of variation for the CCK kit was 10.1% and intra-assay coefficient of variation was 7.9%. GLP-1 levels in plasma samples were measured using a (Total) GLP-1 ELISA kit (EZGLP1T36K, Merck Millipore, Burlington, Massachusetts, USA). Inter-assay coefficient of variation for the GLP-1 kit was 12.5% and intra-assay coefficient of variation was 3.2%. CCK and GLP-1 sample levels were measured according to the manufacturer’s instruction. The microplate reader equipment used for optical density recordings was BMG FLUOstar OPTIMA (BMG Labtech, Mornington, Victoria, Australia).

### Statistical analysis

The Statistical analysis was performed using R software (RStudio, Inc., Boston, Massachusetts, USA). The experimental design consisted of an incomplete Latin square considering “day” and “pig” as blocking criteria (all treatments were tested on every day but not on every pig due to experimental constraints). All data are expressed as the mean ± SEM. Plasma CCK and GLP-1 data were analyzed using the repeated measures function of ANOVA considering “treatment”, “time” and their interaction as fixed effects and “pig” and “day” as random effects. The area under the curve (AUC) was analyzed using a mixed model considering the fixed effect “treatment” and the random effects “pig” and “day”. When significance was found for plasma or AUC data, Dunnett’s post hoc test was performed for the comparison of individual treatments with control. AUC was calculated for each subject/treatment using the trapezoidal rule. Missing data for the calculation of the AUC were estimated using a Best Linear Unbiased Estimator (BLUE) in RStudio. Correlation analysis between plasma CCK and GLP-1 AUC was performed using Pearson’s correlation. The number of samples (*n*) refers to the number of pigs used. Results were considered statistically significant at *P* < 0.05 and tendencies at 0.05 < *P* < 0.1.

## Results

**Figure 2.**
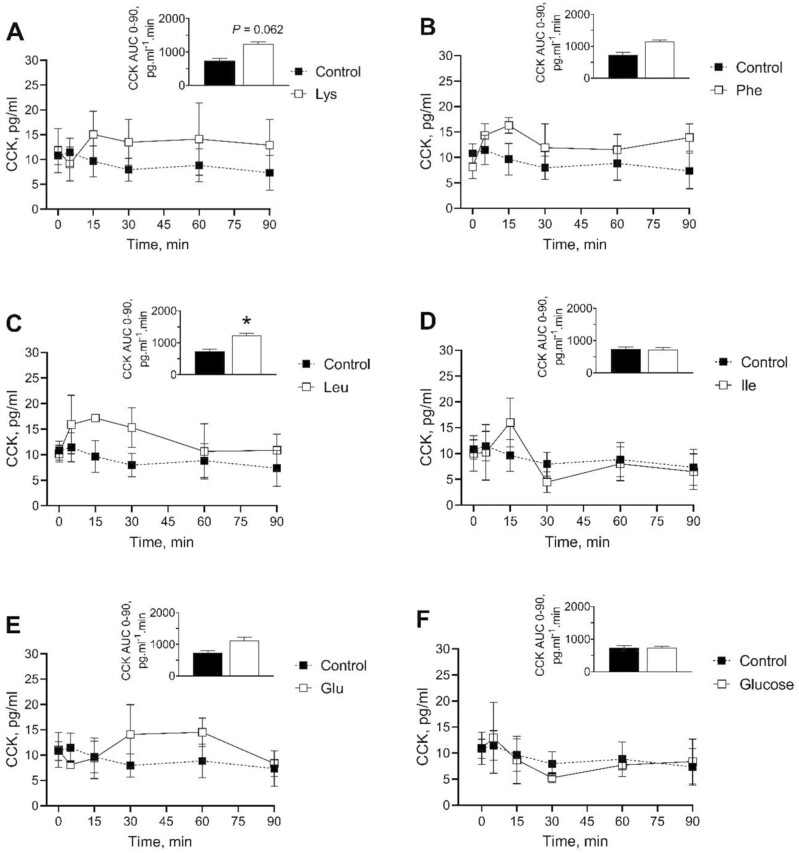
Changes in the blood kinetic of CCK following an oral gavage with AA or glucose in pigs. CCK plasma levels and AUC (0 to 90 min) in young pigs after an oral gavage with water (control) or 3 mmol/kg solutions of Lys (A), Phe (B), Leu (C), Ile (D), Glu (E), or glucose (F). The AUC was derived using a Best Linear Unbiased Estimator (BLUE) to estimate missing data. Baseline values are represented as time 0. Data (CCK plasma levels and AUC) are expressed as the mean + SEM (*n* = 4 or 5). Statistical differences of the AUC between AA treatments and glucose compared to the control are represented as: ^*^P < 0.05.

All pigs had fully recovered from the sedation (behaving and walking normally) within 5 min post-gavage. Figure 2 shows circulating levels of plasma CCK before (baseline represented as time 0 in graphs) and 5, 15, 30, 60 and 90 min after oral gavages of water (control), glucose (positive control), Leu, Ile, Lys, Glu, or Phe. CCK concentrations peaked at 15 min post-gavage in all the AA tested except Glu when the highest concentrations were observed at the 60 min mark. Baseline plasma CCK concentrations did not differ between treatments. Plasma CCK AUC in Leu treated pigs (1236.16 ± 150.26) was significantly (*P* < 0.05) higher than that of the control group (735.63 ± 156.81). In addition, Lys gavaged pigs (1235.42 ± 129.59) tended (*P* < 0.1) to increase CCK AUC compared to the water control group (Figure 2).

**Figure 3.**
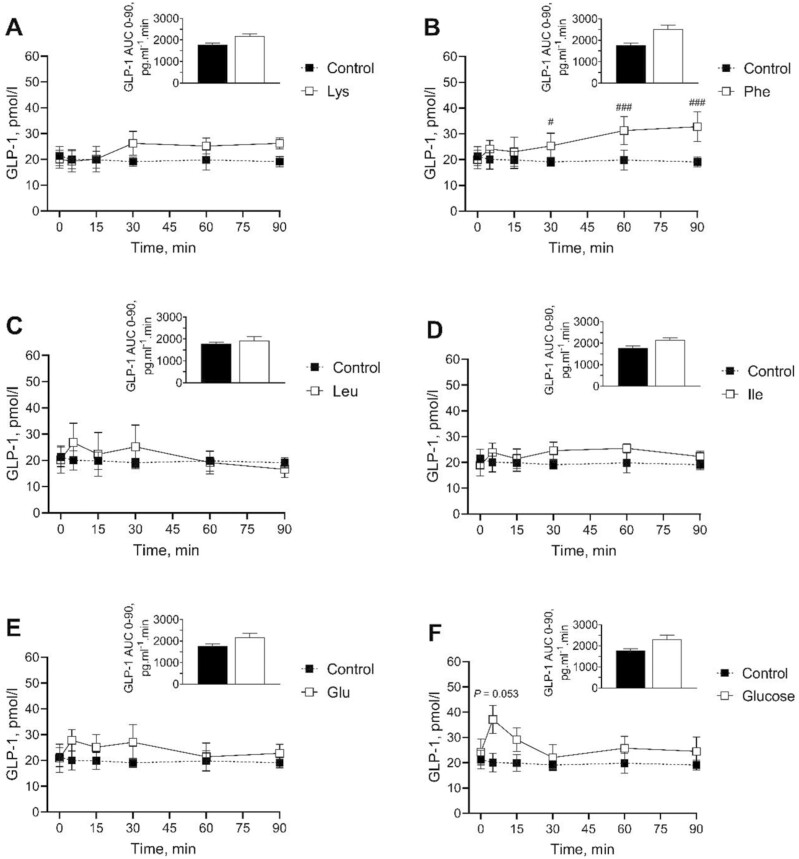
Changes in the blood kinetic of GLP-1 following an oral gavage with AA or glucose in pigs. GLP-1 plasma levels and AUC (0 to 90 min) in young pigs after an oral gavage with water (control) or 3 mmol/kg solutions of Lys (A), Phe (B), Leu (C), Ile (D), Glu (E), or glucose (F). The AUC was derived using a Best Linear Unbiased Estimator (BLUE) to estimate missing data. Baseline values are represented as time 0. Data (GLP-1 plasma levels and AUC) are expressed as the mean + SEM (*n* = 4 or 5). Statistical differences of the GLP-1 values between the AA treatment or Glucose and the baseline are represented as: ^#^*P* < 0.05, and ^##^*P* < 0.001.

Figure 3 shows the GLP-1 plasma concentrations before (baseline depicted as time 0 in graphs) and 5, 15, 30, 60 and 90 min after administering the oral gavages of water (control), glucose (positive control), Leu, Ile, Lys, Glu, or Phe. Baseline plasma GLP-1 concentrations did not differ between treatments. Glucose tended (*P* < 0.1) to rise GLP-1 levels at the 5 min mark (37.13 ± 6.22 pmol/l) when compared to the baseline (24.29 ± 5.12 pmol/l). Phe significantly increased circulating levels of GLP-1 relative to the baseline value (18.43 ± 4.01 pmol/l) at 30 (25.30 ± 4.97 pmol/l; *P* < 0.05), 60 (31.25 ± 5.47 pmol/l; *P* < 0.001) and 90 (32.75 ± 5.75 pmol/l; *P* < 0.001) min post-gavage. It was observed that all the AA except Lys triggered a bi-phasic release of GLP-1, characterized by an early increase 5 min post administration followed by a second increase between 30- and 90-min post-gavage Figure 3. The observation on the bi-phasic pattern has not been statistically supported. No significant differences (*P* > 0.05) on AUC were observed between treatments.

**Figure 4.**
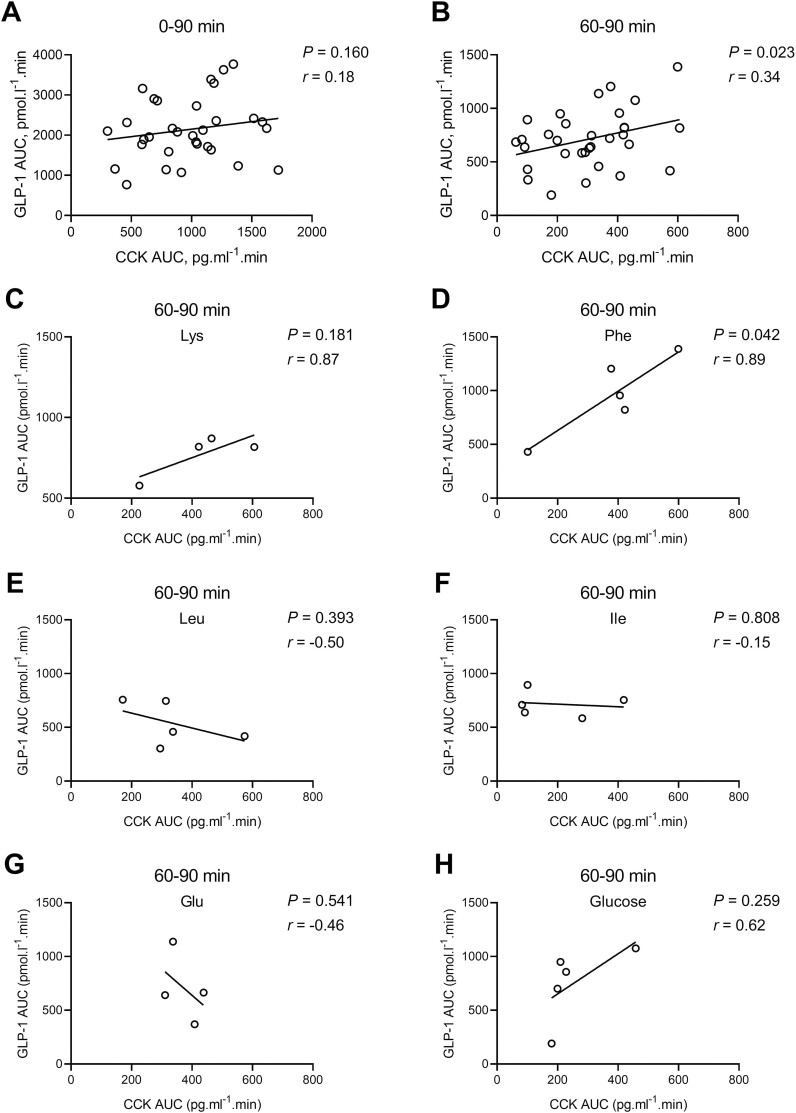
Relationship between plasma cholecystokinin (CCK) and glucagon-like peptide (GLP-1) levels following an oral gavage with amino acids (AA) or glucose in pigs. Relationship between CCK and GLP-1 plasma concentrations (expressed as AUC) for all treatments combined at 0 to 90 min (A) and 60 to 90 min (B) as well as for individual treatments [Lys (C), Phe (D), Leu, (E), Ile (F), Glu (G) and glucose (H)] at 60 to 90 min after an oral gavage in young pigs. The correlation analyses between CCK and GLP-1 data were performed by the Pearson’s correlation test. The *P* < 0.05 was set as the statistical significance.

Correlation analyses between plasma CCK and GLP-1 levels at 0 to 30, 30 to 60, 60 to 90-, and 0 to 90-min post-gavage are shown in Figure 4. Plasma concentrations of CCK and GLP-1 were positively correlated during the 60 to 90 min post-gavage interval (*r* = 0.34, *P* < 0.05). Phe showed a highly significant positive correlation (*r* = 0.89, *P* < 0.05).

## Discussion

As previously reported Leu, Ile, Phe, and Glu stimulated anorexigenic hormone CCK and/or incretin hormone GLP-1 release using a porcine ex vivo model (Müller et al., 2022a). In addition, Lys, Leu, and Ile prolonged satiety after an oral gavage (Müller et al., 2022b). The current study showcased the effect of an oral gavage of Lys, Leu, Ile, Glu, and Phe on the blood kinetic of CCK and GLP-1 in pigs. To our knowledge, this is the first study to investigate the effect of orally gavaged AA on the blood levels of anorexigenic and insulinotropic hormones in live pigs. It was hypothesized that the AA tested would increase plasma CCK and/or GLP-1 levels in a time-dependent manner following an oral gavage.

Leu significantly increased plasma levels of the satiety hormone CCK following the oral gavage. In addition, Lys showed a trend to increase circulating levels of CCK. These findings are relevant for the pig industry since Leu concentrations are commonly high in commercial feeds rich in corn and corn by-products (up to 200% the requirement), and dietary Lys supplementation is a common practice to meet nutritional requirements (Liao et al., 2015, Cemin et al., 2019). The impact of excess dietary Leu inhibiting appetite has been well described in pigs (Wessels et al., 2016; Kwon et al., 2019). Leu significantly reduced feed intake and altered meal patterns prolonging satiety after an oral gavage when administered at concentrations (0.39 g/kg BW) similar to the levels used in the present study (Müller et al., 2022b). The anorexigenic effect of Leu has been previously associated to the release of gut peptides, primarily CCK and GLP-1 from the small intestine under ex vivo conditions (Tian et al., 2019, Müller et al., 2022a). In the present study Leu increased plasma levels of CCK but not of GLP-1, indicating a potentially stronger response from the proximal (where CCK is mainly produced by I cells of the enteroendocrine system) than the distal (the main site of production of GLP-1 by L-cells) small intestine (Tian et al., 2019). Leu showed a less sustained effect on CCK secretion suggesting that the long-term impact on appetite may be partially explained by the release of other gut peptides or post absorptive signals associated with blood branched-chain AA (BCAA) imbalances interacting with the CNS (Lynch et al., 2006; Kwon et al., 2019; Zhang et al., 2019).

In the current study, the impact of Lys on CCK was less significant than the impact of Leu. However, the trend shown by Lys is consistent with the impact of Lys dietary excesses reducing feed intake and growth performance reported by Edmonds and co-workers (1987). In addition, Lys dietary levels have been positively correlated with anorexigenic hormones plasma leptin and intestinal CCK expression levels (Yin et al., 2017). Furthermore, we have also shown that acute oral doses of Lys stimulated early satiation and prolonged satiety in pigs (Müller et al., 2022b). The present results suggest that the anorexigenic effect of Lys were mediated by the secretion of CCK. Compared to the effect reported in Leu, the tendency to increase plasma CCK observed in Lys was delayed indicating that the response might be elicited in proximal and distal regions of the small intestine for Leu and Lys, respectively. These results align with previous reports showing non-significant release of CCK in porcine duodenum together with a higher gene expression of the hormone in jejunum and ileum following the exposure to Lys (Yin et al., 2017; Müller et al., 2022a). In addition, there was a sustained CCK release following the oral gavage of Lys which may explain the lasting impact on appetite described in previous studies (Müller et al., 2022b). The potential role of other hormones with longer half-lifes, such as insulin, on Leu and Lys anorexigenic effects cannot be disregarded (Ren et al., 2007). Furthermore, the stimulation of abdominal vagal afferent fibers and the sensing of increased Leu and Lys blood levels by the CNS could contribute to their satiating effects (Jordi et al., 2013). Overall, the anorexigenic effects of excess Lys or Leu delivered in this research advocate for the implementation of measures to guarantee as much as possible the accuracy in commercial dietary levels of these two AA particularly in post weaning diets. However, the inclusion of moderate excesses of these AA in growing and finisher pig feeds may improve feed efficiency and lean deposition, due to their effect on gut hormone secretion and meal pattern when the requirement for all other EAA are met following the ideal protein profile (Andretta et al., 2016; Müller et al., 2022b).

Phe was the only AA tested that stimulated a highly (*P* < 0.01) significant rise on GLP-1 levels in blood. It did it by following a different pattern than glucose which was used as a GLP-1 secretagogue positive control (Lavin et al., 1998). Glucose has been associated with a rapid increase on GLP-1 following a gavage, compatible with the results obtained in this experiment showing a trend increasing GLP-1 level after only 5 min post-gavage. In contrast, in the current study Phe triggered a comparatively delayed response passed 30 min of oral delivery, which was sustained until the final measuring time of 90 min. Prolonged increases on GLP-1 have been illustrated when nutrients come in direct contact with L-cells in the ileum and colon (Wu et al., 2015). However, previous data showed no effect of Phe on GLP-1 secretion or feeding behavior (Müller et al., 2022a, 2022b). Thus, the present results indicate that Phe may primarily elicit an incretin function affecting glucose absorption and metabolism rather than a direct anorexigenic effect in pigs. O’Connor and co-workers (2003) discovered that the dietary supplementation of Phe had the potential to improve muscle growth via the GLP-1 mediated incretin effect (O’Connor et al., 2003). Considering the effect of Phe on GLP-1, the aromatic AA may also modulate glucagon and insulin circulating levels(Gannon and Nuttall, 2010). However, additional studies investigating the effect of Phe on insulin and incretin hormones are required to confirm if this is the case in pigs. In addition, Phe is an essential precursor of catecholamines such as epinephrine, and norepinephrine involved in gut smooth muscle contraction and potentially the ileal break (Pashaei et al., 2022).

The molecular link between dietary Phe, gut hormone secretion and satiety appears to be the calcium-sensing receptor (CaSR) in lab rodents (Conigrave and Brown, 2006). CaSR is an extracellular nutrient sensor expressed abundantly throughout the gastrointestinal tract that responds to aromatic AA, including L-Phe and L-Trp, among other nutrients such as Ca^2^ ions. The release of gut peptides has been associated to a network of enteroendocrine cells that respond to dietary nutrients through the stimulation of receptors present in the apical membrane of the cells (Roura and Navarro, 2018, Müller et al., 2021). The transmembrane receptor gene repertoire in pigs have been described and those related to AA or peptide sensing identified including the CaSR (Roura et al., 2011; Da Silva et al., 2014; Roura and Fu, 2017). Current evidence indicates a mediating role of CaSR on aromatic AA-induced CCK release in the porcine ileum (Feng et al., 2019). Leu, Ile, and Glu had previously been shown to release GLP-1 under ex vivo conditions in pigs (Müller et al., 2022b). In contrast, in the present study these three AA did not affect plasma GLP-1. The high catabolism rate of BCAA and Glu by enterocytes in the small intestine may have contributed to insufficient levels of these AA reaching the distal gut where the highest release of GLP-1 occurs (Janeczko et al., 2007; Chen et al., 2009). Except for the T1R1-T1R3 dimer little is known on the presence and function of AA receptors in the porcine gut. Thus, the molecular links involved in gut peptide secretion in pigs warrants further investigation.

While the direct effect of Phe on CCK was not significant, a strong correlation between CCK and GLP-1 plasma concentrations was identified. Given that CCK and GLP-1 are primarily released in duodenum and in ileum, respectively, the correlation suggests that Phe-induced GLP-1 released in the distal gut stimulates CCK secretion in proximal segments via an unknown feedback mechanism in pigs. It has been suggested that the stimulation of vagal afferents may result in feedback mechanisms in the gut (Rocca and Brubaker, 1999). In addition, the ileal brake triggered by GLP-1 released in the ileum and colon may facilitate CCK secretion by slowing the transit of nutrients in the proximal gut (Maljaars et al., 2007). Overall, the results obtained with the Phe gavage depicted a complex scenario that resulted on a sustained long-term increase in circulating GLP-1. Several mechanisms may contribute to explain the results including gut peptide-mediated feedback mechanisms, interactions with glucose digestion and absorption, and the release of catecholamines. However, most of these mechanisms lack experimental validation in pigs.

On a final note, it is acknowledged that the results of this research were obtained using to non-protein bound and single AA delivered by oral gavage by-passing the oral cavity into the stomach. However, the effect of AA as part of a meal (protein bound or free) on gut hormones may be altered by the delivery system and by nutrient interactions what will require further investigations.

## Conclusions

Leu and to a lesser extend Lys triggered the release of the anorexigenic hormone CCK after an oral gavage in pigs. Phe-induced the secretion of the incretin hormone GLP-1 following a delayed release pattern when compared to the early peak (at 5 min post-gavage) observed in glucose. The increase in GLP-1 plasma levels as a response to Phe took place 30 min after the dose and continued beyond the timeline registered in this experiment which was of 90 min. In addition, a positive correlation between CCK and GLP-1 levels in blood was observed in Phe treated animals suggesting the existence of a feedback mechanism between the proximal (releasing CCK) and the distal (releasing GLP-1) small intestine. Our observations provide new insights on the impact of dietary free AA on gut peptide secretion and appetite highly relevant to feed formulation practices in pigs.

## Abbreviations

AA: amino acid
AUC: area under the curve
BCAA: branched-chain amino acids
CaSR: calcium-sensing receptor
CNS: central nervous system
CCK: cholecystokinin
EEC: enteroendocrine cells
GIT: gastrointestinal tract
GLP-1: glucagon-like peptide 1
